# Dysphagia Onset in Older Adults during Unrelated Hospital Admission: Quantitative Videofluoroscopic Measures

**DOI:** 10.3390/geriatrics3040066

**Published:** 2018-10-03

**Authors:** Marie Jardine, Anna Miles, Jacqui Allen

**Affiliations:** 1 Speech Science, The University of Auckland, Auckland, 1142, New Zealand; a.miles@auckland.ac.nz; 2 Waitemata District Health Board, Auckland 0740, New Zealand; jeallen@voiceandswallow.co.nz; 3 Department of Surgery, The University of Auckland, Auckland 1142, New Zealand

**Keywords:** dysphagia, older age, swallowing, videofluoroscopy, hospitalization, frailty, sarcopenia

## Abstract

New-onset swallowing difficulties in older patients during unrelated hospital admissions are well recognized and may result in prolonged hospital stay and increased morbidity. Presbyphagia denotes age-related swallowing changes which do not necessarily result in pathological effects. The trajectory from presbyphagia to dysphagia is not well understood. This retrospective observational study compared quantitative videofluoroscopic measures in hospitalized older adults aged 70–100 years, reporting new dysphagia symptoms during admission (*n* = 52), to healthy asymptomatic older (*n* = 56) and younger adults (*n* = 43). Significant physiological differences seen in hospitalized older adults but not healthy adults, were elevated pharyngeal area (*p* < 0.001) and pharyngeal constriction ratio (*p* < 0.001). Significantly increased penetration (*p* < 0.001), aspiration (*p* < 0.001) and pharyngeal residue (*p* < 0.001) were also observed in the hospitalized older cohort. Reasons for onset of new swallow problems during hospitalization are likely multifactorial and complex. Alongside multimorbidity and polypharmacy, a combination of factors during hospitalization, such as fatigue, low levels of alertness, delirium, reduced respiratory support and disuse atrophy, may tip the balance of age-related swallowing adaptations and compensation toward dysfunctional swallowing. To optimize swallowing assessment and management for our aging population, care must be taken not to oversimplify dysphagia complaints as a characteristic of aging.

## 1. Introduction

Successful aging is a process of adaptation [1]. Aging involves a kaleidoscope of physical, psychosocial, environmental, and disease-related factors. Medical advancements have contributed to increased life expectancy and our globally aging population. Age can be just a number until age-related changes coincide with emergence of disease states or deconditioning. This then tips the balance and outstrips physiologic reserve, often resulting in rapid decline. Social events play an essential role in enjoyment for older adults, typically centered around eating and drinking. Therefore, dysphagia (swallowing difficulties) can lead to serious social and emotional consequences [2], in addition to increased risk of dehydration, malnutrition [3] and aspiration pneumonia [4]. Well known reasons for swallowing dysfunction are stroke [5], traumatic brain injury [6], progressive neurological disease [7,8,9] and head and neck cancer [10]. While the increasing prevalence of swallowing problems in older age is highlighted in the literature, aging itself is not a causative factor. Age-related swallowing change that preserves function and safety is termed presbyphagia. However, what tips the balance from presbyphagia to swallowing impairment is unclear.

Efficient swallowing requires coordinated constriction, expansion and displacement of pharyngeal structures for bolus propulsion through the pharynx and upper esophageal sphincter, while the airway is protected [11]. Oral adaptations with age are self-evident, such as loss of dentition and saliva [12]. Pharyngeal and esophageal changes in timing and displacement are well documented [13,14]. Over time, older adults make changes independently by taking smaller mouthfuls, metering the dose, and avoiding challenging food textures. An acute event or illness may destabilize these adaptations, resulting in swallowing difficulties in older age, due to reduced functional reserve, sarcopenia, comorbidities and polypharmacy, which individually or together challenge homeostasis [15]. This is reflected in rising rates of hospital referrals for swallowing assessments for older patients. From 2007–2014, a study documented an increase of 63% in acute speech-language therapy referral rates for ≥60-year-olds, including a 222% increase for patients 90 years and older [16]. More recently, in older hospitalized adults without history of swallowing-related disease, 26% were identified with elevated Eating Assessment Tool (EAT-10) [17] scores suggestive of dysphagia risk, and this was associated with poorer functional recovery [18].

A period of physical inactivity during hospitalization is known to compromise functional capacity [19]. Prolonged immobility correlates with slower recovery, longer length of stay and increased hospital costs [20]. Poor biological reserve limits the ability to endure and recover from a period of illness [21]. Furthermore, a minor insult may exacerbate chronic illness or functional decline [22]. The prevalence of frailty in hospitalized older adults was recently estimated between 50–87% [23]. Frailty is complex because it is multidimensional, comprising of physical, psychological, social, and nutritional factors. This leads to ‘confusion as to which occurs first—frailty or the factor’ [24] (p. 288). Malnutrition in older adults is a marker of frailty, stemming from a combination of medical and social factors, rather than the acute reason for admission [25].

Clinical questions remain: why do older patients develop dysphagia during their hospital admission? Is there a commonality of swallowing physiological changes for these patients? The primary aim of this retrospective observational study was to investigate older hospitalized adults who developed dysphagia during their admission, through quantitative videofluoroscopic study of swallowing (VFSS) measures [14]. The secondary aim was to compare swallowing timing and displacement measures between hospitalized older adults, healthy older adults, and healthy younger adults. It was hypothesized that hospitalized older adults would demonstrate more swallowing variability compared to healthy older and younger adults. Furthermore, it was also hypothesized that hospitalized older adults would demonstrate increased penetration or aspiration and pharyngeal residue compared to healthy older and younger adults.

## 2. Materials and Methods

This retrospective observational study received appropriate, local ethical approval: VFSS database (University of Auckland Human Participants Ethics committee: 9263) and normative database (New Zealand Health and Disability Ethics Committee: 13/STH/202).

### 2.1. Participants

VFSS of patients aged 70 years and older performed between May 2013–June 2018 were retrospectively reviewed. Patients’ past clinical letters were screened for study inclusion criteria: hospitalized with no history of dysphagia or diagnosis known to affect swallowing, such as acute neurological event, dementia or head and neck cancer. Patients who met the study’s criteria were included consecutively in the study. Demographics, comorbidities, medications, esophageal screening or investigation, time from admission until VFSS, length of stay and mortality were collected from clinical letters and VFSS reports. Clinical letters were also reviewed for information on nutrition (weight loss, dietitian input during hospital stay, community dietitian follow up, or recommended weight monitoring by General Practitioner).

**Figure 1:**
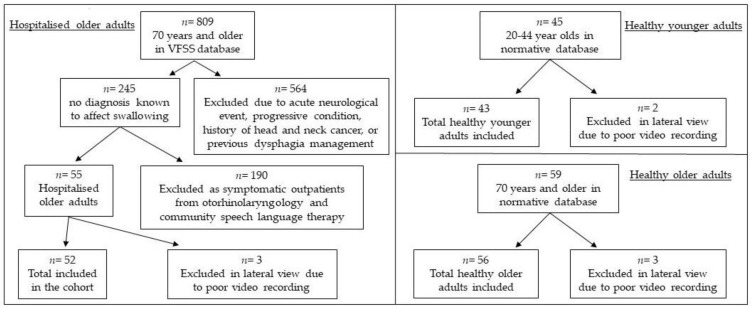
Reasons for lateral video exclusions in each cohort: hospitalized older adults, healthy younger adults, and healthy older adults.

Between April–November 2014 and August–September 2017, 139 healthy volunteers without history of dysphagia or diagnosis known to affect swallowing were recruited and underwent VFSS at North Shore Hospital in Auckland. Participant responses to the Functional Oral Intake Scale (FOIS) [26], EAT-10 [17], Sydney Swallow Questionnaire (SSQ) [27] and Mini-Nutritional Assessment (MNA) [28] were within the normal range. For the current study, participants were divided into two groups: healthy younger adults (age < 70 years) and healthy older adults (age ≥ 70 years). Reasons for excluded videos (VFSS) in lateral view are detailed for each cohort in Figure 1. Twenty-five percent of esophageal screens for hospitalized older adults were excluded due to missing data (15.4%), poor recording (5.8%) and no thin liquid trialed (3.8%). One esophageal screen for healthy younger adults and two esophageal screens for healthy older adults were excluded due to poor recording.

### 2.2. Procedure

A standardized VFSS protocol was used at the research site for both patients and healthy controls, similar to the Leonard and Kendall protocol [14]. VFSS were performed in a radiology suite using a Videofluoroscope (Toshiba, Tokyo, Japan) and recorded at 30 frames per second directly onto USB drive in AVI format. A 2 cm diameter radiopaque ring was placed under the chin for calibration during analysis. Participants were recorded in standing or seated position. In anteroposterior view, participants swallowed 20 mL liquid barium 100% *w*/*v*/56% *w*/*w* suspension (Liquid Polibar diluted with water), 3 mL barium paste 60% *w*/*w* (E-Z-paste) and a 13 mm round barium pill. In lateral view, participants swallowed 1 mL, 3 mL, 20 mL liquid barium, 100 mL liquid barium sequential straw drinking and 3 mL barium paste.

### 2.3. Measures

For this study, data was investigated from 20ml liquid barium in anteroposterior and lateral views. In anteroposterior view, esophageal transit times were measured using a timing component on Swallowtail (Belldev Medical), which is a software platform to perform quantitative swallowing timing and displacement analyses [14]. In lateral view, videos were analyzed frame by frame on Swallowtail (Belldev Medical); the measures are detailed in Table 1. Swallows were rated against the Penetration-Aspiration Scale [29] and residue was calculated using the Bolus Clearance Ratio [30]. Number of swallows per bolus were also observed.

### 2.4. Analysis

Statistical analyses were completed using SPSS (Version 25). Quantitative timing and displacement data were screened for parametric assumptions. Normality was assessed using the Kolmogorov-Smirnov test and by visualizing frequency distributions of the dependent variables. PESdur (PES opening duration), PESmax (maximum distension of the PES) and PAhold (pharyngeal area at rest) were normally distributed (*p* > 0.05), therefore mean (*M*) and standard deviation (*SD*) were reported, and differences between the hospitalized and healthy groups were tested using Analysis of Variance (ANOVA). Post-hoc Bonferroni test was performed on significant results (a priori *p* < 0.05) to determine differences between groups. Most of the timing and displacement measures demonstrated high skew and kurtosis, violating parametric assumptions. Accordingly, median (*Mdn*) and interquartile range (*IQR*) were considered to demonstrate a better dispersion of the nonparametric data. Groups were compared using the Kruskal-Wallis Test. For significant results (a priori *p* < 0.05), post-hoc pairwise comparisons with adjusted *p*-values were performed and effect sizes (*r*) were calculated [31]. Categorical data was reported as counts and percentages and Chi-square tests were performed to assess differences between groups. The association between length of hospital stay, time from admission until VFSS and quantitative measures were tested using Spearman’s correlation coefficient.

## 3. Results

Participant demographics are presented in Table 2. Characteristics of hospitalized older adults are presented in Table 3. Medical reasons for hospitalizations included pneumonia (9), cardiac (7), general unwellness (6), abdominal (4), urinary (3), renal (2) and depressive (2). Surgical reasons for hospitalization were fall (15) and elective (4). Forty-nine (94.2%) older adults admitted to hospital presented with five or more comorbidities and 47 (90.4%) were discharged on five or more medications. Length of hospital stay was less than 10 days for 12 patients (23.1%) and 10 or more days for 40 patients (76.9%). Two patients passed away during admission and six patients were excluded from mortality rate as the time of VFSS was less than 2 months before data collection.

### 3.1. Quantitative Swallowing Measures

Timing and displacement measures across the three cohorts are presented in Table 4: healthy younger adults, healthy older adults and hospitalized older adults. Post-hoc comparisons of statistically significant measures are presented in Table 5, to determine significant differences between groups. Non-normally distributed timing and displacement measures that were significantly different between the older cohorts were compared using the *IQR* of healthy older adults. For hospitalized older adults, 64.6% of PCR (pharyngeal constriction ratio), 66.0% of HLmax (hyoid to larynx maximum approximation) and 83.0% of BCR (bolus clearance ratio) sat above the *IQR* of healthy older adults. As PAhold (pharyngeal area) followed a normal distribution, measures for hospitalized older adults were compared using the *SD* of healthy older adults; 55.1% were above one *SD*.

### 3.2. Subjective Ratings

#### 3.2.1. Penetration-Aspiration Scale

Penetration-Aspiration Scale (PAS) ratings were significantly different between groups, H(2) = 36.41, *p* < 0.001. Pairwise comparisons with adjusted *p*-values showed PAS ratings for hospitalized older adults were significantly higher compared to healthy younger (*p* < 0.001, *r* = 0.44) and older adults (*p* < 0.001, *r* = 0.41) (healthy younger adults *Mdn* = 1, *IQR* = 0, spread = 1; healthy older adults *Mdn* = 1, *IQR* = 0, spread = 1–3; hospitalized older adults *Mdn* = 1, *IQR* = 2, spread = 1–8). No penetration was observed in healthy younger adults. A PAS score of 2 or 3 was rated in three (5.4%) healthy older adults and in 14 (26.9%) hospitalized older adults. No aspiration was detected in healthy younger or older adults. A PAS score of 5 (aspiration) or more was rated in seven (13.4%) hospitalized older adults. There was no significant difference in PAS ratings between younger and older adults (*p* = 1.00, *r* = 0.06).

#### 3.2.2. Number of Swallows Per 20 mL Bolus

Number of swallows for a thin liquid 20ml bolus significantly differed between cohorts, H(2) = 28.20, *p* < 0.001. Pairwise comparisons with adjusted *p*-values showed that compared to younger controls, significantly more swallows were performed by healthy older (*p* = 0.002, *r* = 0.27) and hospitalized older adults (*p* < 0.001, *r* = 0.43) (healthy younger adults *Mdn* = 1, *IQR* = 0, spread = 1–2; healthy older adults *Mdn* = 2, *IQR* = 0, spread = 1–4; hospitalized older adults *Mdn* = 2, *IQR* = 1, spread = 1–6). Per 20 mL bolus, 43.0% of healthy older adults swallowed more than once, while 59.6% of hospitalized adults required more than one swallow per bolus, which was not significantly different (*p* = 0.092, *r* = 0.18).

### 3.3. Length of Stay

There was no correlation between quantitative timing and displacement measures and length of stay. There was a significant association between length of stay and time from admission until VFSS, *r*_s_ = 0.614, *p* < 0.001. The later the VFSS was performed from admission, the longer the length of hospital stay.

## 4. Discussion

This retrospective observational study presents quantitative videofluoroscopic measures of new-onset dysphagia during unrelated medical or surgical admissions in older adults, aged 70–100 years old. Greater impairment and more variability in swallowing measures according to *IQR* were observed in hospitalized older adults, which confirms our first hypothesis. When comparing quantitative measures, pharyngeal area, PCR and BCR were statistically different between hospitalized older adults and both healthy adult groups. Hospitalized older adults demonstrated significantly increased penetration, aspiration, and pharyngeal residue. This is in concordance with our second hypothesis. Differences observed between hospitalized older adults and healthy adults may indicate impairment (pharyngeal area, PCR, penetration/aspiration, and BCR), whereas differences between the healthy younger and older adults are likely age-related changes (total pharyngeal transit time and esophageal transit time). While we cannot infer causation of dysphagia from VFSS in this retrospective study, we will consider possible reasons for swallowing changes, including the impact of hospitalization on older adults.

Our hospitalized older cohort were not admitted with swallowing difficulties, but rather developed these problems later in admission, with VFSS occurring up to 75 days after admission. This is considerably longer than studies reporting referral timing following acute stroke [32] or intensive care admission [33] where swallowing difficulties are directly related to the reason for admission. After four days of bed rest, effects of disuse in limb muscle fibers for older adults include reduced muscle strength, force and function [34]. After ten days of bed rest for a healthy cohort, aerobic capacity and lower extremity muscle strength were reduced [35]. Patients made nil by mouth (NBM) may experience a similar phenomenom during swallowing, with disuse of pharyngeal constrictors and suprahyoid muscles contributing to difficulty swallowing [36]. Increased multimorbidity and polypharmacy in older patients may further impact functional reserve during serious illness. In our hospitalized cohort, 94% presented with five or more comorbidities and 90% took five or more medications. A recent study performed at the same research site, recruited a general sample of hospitalized adults 85 years and older [37]. They also identified a high proportion of patients with five or more comorbidities (77%) and taking five or more medications (71%), which suggest a commonality of multimorbidity and polypharmacy in these older patient groups.

A host of factors during hospitalization for older adults likely contribute to new-onset of swallowing impairment. The PCR is considered a valid surrogate measure for manometric pharyngeal pressure [38]. Maximum pharyngeal constriction for healthy adults is approximately zero, as was observed in our healthy adults (with older age, this may extend up to 0.14 cm^2^ [14]). Elevated (worse) PCR was identified in our hospitalized older adults, which may reflect weak tongue base propulsion and/or poor pharyngeal constriction. Larger pharyngeal space, perhaps due to atrophy of pharyngeal muscles [39] or laryngeal descent, is another consideration. Increased pharyngeal area has been reported in advancing age [40] and increased pharyngeal volume has been associated with worse pharyngeal constriction in healthy older adults [41]. Incomplete pharyngeal constriction is associated with post-swallow pharyngeal residue [39] and the presence of pyriform residue has been attributed to reduced pharyngeal shortening [42]. There was no significant difference in PCR or BCR between healthy younger and older adults in the present study. The elevated PCR and BCR in hospitalized older adults may therefore be considered pathologic, with incomplete pharyngeal clearance indicating either pharyngeal weakness and/or outlet obstruction.

Prolonged pharyngeal and esophageal transit times were observed in hospitalized and healthy older adults compared to younger adults. Increased transit times with age reflects previous studies [13,14,42]. Pharyngeal bolus transit is considered a primary marker of ‘pharyngeal health’ [43]. Age-related increased pharyngeal transit times may be attributed to decreased motor unit firing rates (especially when force is exerted) and slower contractile properties [44], characterized by reduced pressure generation and weaker contractions [45]. More hospitalized older adults demonstrated longer pharyngeal transit times than healthy older adults. No aspiration and minimal penetration events were detected in healthy adults, which supports that age-related changes do not compromise airway protection. Some healthy older adults metered the bolus or swallowed again to clear oral residue. This perhaps offers insight into overall swallowing effort or stress on airway protection [46], which may reach a tipping point during a serious illness in hospital.

The mean age of the hospitalized older group was 85 years old (*SD* = 7). A recent local study on prevalence of dysphagia and malnutrition in hospitalized adults 85 years and older identified 30% with dysphagia risk using the EAT-10, 30% malnourished and 43% at risk of malnutrition using the Multi-nutritional Assessment (MNA) [28,37]. During consecutive data selection of patients aged 70 years and older in the current study, the majority were over 80 years old (69%). This may reflect heightened vulnerability to swallowing problems during serious illness in advanced age due to reduced functional reserve. In a study of functional decline post-hospitalization, over half of patients aged 85 years and older had worse function in activities of daily living than their pre-illness baseline, compared to one quarter of 70–74 year old patients [47].

The median length of hospital stay was 20 days; the maximum was 135 days. A study reported that older patients with new dysphagia almost tripled their length of stay in the intensive care unit (ICU) and doubled their time in hospital. These older patients also experienced more complications during admission and a lower rate were discharged home [33]. A higher level of care is synonymous with frailty in older adults. The relationship between frailty and hospitalization is bidirectional; not only does frailty increase the risk of hospitalization, the period of acute care also contributes to worsening frailty [48].

Nutritional frailty involves sarcopenia, unintentional weight loss and disability, often foreshadowing terminal decline [15]. It is likely that our hospitalized cohort were at risk of malnutrition, yet only one third of discharge reports detailed nutritional support in hospital or arranged for follow up in the community. Use of nutrition and dysphagia screening tools may be a way to identify those at risk early in an admission, bringing support and input prior to development of secondary pathology [15,18,37]. Dietetic and speech-language therapy review can provide specific dietary advice and strategies for eating and drinking.

Several questions about the hospitalized cohort remain unanswered due to the retrospective design of this study. Pre-hospital functional level for the hospitalized adults was not available. We were reliant on clinical and VFSS reports, which may be incomplete. Secondly, while frailty and sarcopenia were referred to in this study, data on prevalence or measurement of frailty or sarcopenia were unavailable. Similarly, dentition, weight, BMI, delirium, level of fatigue and functional status during admission were unknown. The patient cohort was from a single hospital possibly limiting generalization of results. Furthermore, consecutive data selection resulted in an imbalance of males and females.

The impact of hospitalization on gross motor function is well researched. However, due to differences in muscle composition between limbs and the swallowing mechanism [49], the application of hospitalization to swallowing is not fully understood. Our globally aging population calls for more research in this area. The relationship between swallowing and functional status pre-admission, during hospitalization and post-discharge should be explored. With growing evidence of the impact of sarcopenia on dysphagia, future prospective studies on older adults should measure frailty and sarcopenia compared to dysphagia risk or quantitative swallowing measures. For those at risk of frailty and sarcopenia, predictive factors for swallowing impairments should be investigated. More research is needed regarding screening protocols, preventative interventions, and rehabilitation for older adults with swallowing difficulties with no primary cause. We need to continue to distinguish age-related conditions from aging processes, to prevent undermanaging or overmanaging older patients.

## 5. Conclusions

This study presents quantitative videofluoroscopic study of swallowing analyses of hospitalized older adults with acute onset dysphagia during unrelated medical or surgical admissions. Comparison to non-hospitalized healthy adults revealed significant physiological differences in pharyngeal area and PCR, implicating pharyngeal weakness in symptom production. Reasons for new-onset of swallowing problems in the hospitalized cohort are likely multifactorial and complex. Critical illness involves muscular changes in structure and function [50], and older adults are less resilient or able to return to baseline [51] due to frailty and sarcopenia. This may be accelerated by physical inactivity and poor nutrition during hospitalization [19]. It is important to raise awareness of prevalence and risk of swallowing dysfunction and malnutrition in older age. Screening should be standard practice. Attributing dysphagia symptoms as age-related change is misinformed. To optimize dysphagia assessment and management, care must be taken not to oversimplify dysphagia as a characteristic of aging.

## Figures and Tables

**Table 1:** Quantitative swallowing measures of timing, displacement, and area ratio.

Measure	Description
Timing (sec)	
Hdur	Duration of maximum hyoid displacement
PESop	Pharyngoesophageal segment (PES) opening duration
Airwaycl	Onset and completion of supraglottic closure
Airwaydur	Duration of airway closure
BP1AEcl	Time from airway closure to bolus entering PES
TPT	Onset of swallow (first movement past the posterior nasal spine) to clearance of the bolus tail through the PES: total pharyngeal transit time.
ETT	Entrance of the bolus through the PES to clearance through the lower esophageal sphincter (LES): esophageal transit time
Displacement (cm)	
PESmax	The maximum distension of the PES
Hmax	Change in hyoid position from rest to maximum anterior-posterior displacement
HLmax	Difference in distance between hyoid and larynx at rest and when maximally approximated during swallow
Area (cm^2^)	
PAhold	Area of pharynx at rest
Ratio (area:area)	
PCR	Pharyngeal constriction ratio: pharyngeal area of maximum constriction/ open pharyngeal area
BCR	Bolus clearance ratio: bolus residual/ area of bolus prior to PES opening

**Table 2:** Demographics of the participants.

*n*		Healthy Younger Adults	Healthy Older Adults	Hospitalized Older Adults	*p*-Value
		43	56	52	
Age (years)	*M*, *SD* *Range*	30.84, 7.71 20–44	81.20, 8.18 70–99	84.73, 7.02 71–100	<0.001
Sex *n* (%)	Female Male	24 (55.8%) 19 (44.2%)	35 (62.5%) 21 (37.5%)	18 (34.6%) 34 (65.4%)	0.011
Ethnicity *n* (%)	NZ Māori NZ European Other	4 (9.3%) 24 (55.8%) 15 (34.9%)	1 (1.8%) 49 (87.5%) 6 (10.7%)	1 (1.9%) 42 (80.8%) 9 (17.3%)	0.004
Residence *n* (%)	Independent Rest home	43 (100%) 0	45 (80.4%) 11 (19.6%)	46 (88.5%) 6 (11.5%)	0.009

**Table 3:** Characteristics of hospitalized older adults.

	*Mdn*	*IQR*	Spread
Comorbidities	9	5	1–20
Medications	10	6	2–19
Days admitted until VFSS	15	25	2–75
Length of stay (days)	21	47	3–135
	*n* (%)		
Reason for admission	Medical 33 (63.5%) Surgical 19 (36.5%)		
ETT documented ^1^	Yes 35 (67.3%) No 17 (32.7%)		
Nutrition documented ^2^	Yes 16 (32.7%) No 36 (67.3%)		
Mortality since VFSS	<2 months 10 (19.2%) 2–6 months 8 (15.4%) 6–12 months 1 (1.9%) 2–3 years 4 (7.7%) Living 23 (44.2%)		

^1^ ETT—Esophageal transit time. Esophageal screen or investigation was referred to in clinical or VFSS report. ^2^ Information on nutrition was detailed in discharge report.

**Table 4:** Characteristics of hospitalized older adults.

Measure ^1^	Healthy Younger Adults		Healthy Older Adults		Hospitalized Older Adults		*p*-Value
	*Mdn*	*IQR*	*Mdn*	*IQR*	*Mdn*	*IQR*	
Hdur	0.30	0.19	0.27	0.15	0.33	0.17	0.291
PESop ^2^	0.60	0.07	0.62	0.11	0.61	0.17	0.732
Airwaycl	0.20	0.30	0.27	0.30	0.30	0.53	0.028
Airwaydur	0.80	0.22	0.87	0.37	0.80	0.47	0.221
BP1AEcl	−0.07	0.07	−0.15	0.23	−0.07	0.30	<0.001
TPT	0.77	0.13	0.90	0.23	1.00	0.60	<0.001
ETT	5.58	4.18	7.97	11.02	10.58	11.33	<0.001
PESmax ^2^	0.78	0.15	0.77	0.19	0.72	0.21	0.384
Hmax	1.81	0.47	1.86	0.75	1.53	0.51	0.023
HLmax	0.94	0.31	0.63	0.27	1.07	1.00	<0.001
Pahold^2^	11.28	3.07	11.45	2.51	14.35	3.12	<0.001
PCR	0.01	0.00	0.02	0.02	0.06	0.06	<0.001
BCR	0.00	0.00	0.00	0.00	0.054	0.07	<0.001

^1^ refer to Table 1 for definitions of measures. ^2^ *M* and *SD*.

**Table 5:** Post-hoc comparisons between hospitalized older adults and healthy adults, and healthy older and younger adults.

		Healthy Younger Adults		Healthy Older Adults			Healthy Younger Adults	
Hospitalized older adults	Measure ^1^	*p*	*r*	*P*	*r*	Healthy older adults	*p*	*r*
	Airwaycl	0.026 *	0.22	1	0.02		0.167	0.16
	BP1AEcl	1	0.02	0.004 *	0.26		0.011 *	0.24
	TPT	<0.001 *	0.45	0.120	0.17		0.001 *	0.30
	ETT	<0.001 *	0.38	0.158	0.17		0.013 *	0.24
	Hmax	0.198	0.15	0.023 *	0.22		1	0.06
	HLmax	0.410	0.12	<0.001 *	0.47		<0.001 *	0.32
	PAhold	<0.001 *	-	<0.001 *	-		1	-
	PCR	<0.001 *	0.48	<0.001 *	0.50		1	0.02
	BCR	<0.001 *	0.61	<0.001 *	0.76		0.627	0.11

^1^ refer to Table 1 for definitions of measures. * statistically significant, *p* < 0.05.
